# Review of the genus *Laelaspisella* Marais & Loots, with the description of a new species from Iran (Acari, Laelapidae)

**DOI:** 10.3897/zookeys.549.6939

**Published:** 2016-01-05

**Authors:** Omid Joharchi, Esmaeil Babaeian, Alireza Jalalizand

**Affiliations:** 1 Department of Plant Protection, Yazd Branch, Islamic Azad University, Yazd, Iran; 2 Department of Plant Protection, Faculty of Agriculture, University of Tehran, Iran, Karaj; 3 Department of Entomology, Collage of Agriculture, Khorasgan Branch, Islamic Azad University, Isfahan, Iran

**Keywords:** *Laelaspisella*, Laelapidae, elm trees, Isfahan, Pseudoparasitus (Gymnolaelaps) tonsilis Karg, Iran

## Abstract

A new species of mite is described from Iran, *Laelaspisella elsae* **sp. n.** (Acari: Laelapidae). The new species was collected from bark of elm trees in Isfahan province. A revised diagnosis for *Laelaspisella*, as well as a key to the world species of the genus, are presented. Two species groups of *Laelaspisella* are proposed: those with seta *pd3* on genu I and those without *pd3* on genu I. Pseudoparasitus (Gymnolaelaps) tonsilis Karg, 1989a is transferred to *Laelaspisella*, based on its hypertrichous holodorsal shield, metasternal setae *st4* absent and genu IV with ten setae. The problems with *Laelaspisella canestrinii* are explained and *Laelaspisella canestrinii* *sensu* Berlese (1903), (1904) and Costa (1962) is provided with a new name, *Laelaspisella berlesei* Joharchi, **nom. n.**

## Introduction

The genus *Laelaspisella* Marais & Loots, 1969 was described for two species found in soil in southern Africa. *Laelaspisella* was considered as a subgenus of *Hypoaspis* *sensu lato* by Karg (1989b), who also included Hypoaspis (Laelaspisella) foramenis Karg, 1989b and Hypoaspis (Laelaspisella) cavitatis Karg, 1982 in this genus. Then Karg (2013) considered *Laelaspisella* as genus of Hypoaspidinae Vitzthum, *sensu* Karg (2000) and regarded this genus as a sister genus of *Pneumolaelaps* Berlese.

The most recent taxonomic work on the genus was by Joharchi and Halliday (2013), who clarified the diagnosis of genus *Laelaspisella*, and transferred *Gymnolaelaps kabitae* Bhattacharyya, 1968 and *Gymnolaelaps canestrinii* (Berlese) *sensu* Costa, 1962 to *Laelaspisella*, and excluded the two species described by Karg from *Laelaspisella*. Before the present study, only four species of *Laelaspisella* had been reported, *Laelaspisella macrodorsalis* Marais & Loots, 1969; *Laelaspisella epigynialis* Marais & Loots, 1969; *Laelaspisella canestrinii* (Berlese) *sensu* Costa and *Laelaspisella kabitae* (Bhattacharyya). A further new species is described and a key is presented for the identification of *Laelaspisella* species. One species is transferred from Pseudoparasitus (Gymnolaelaps) to *Laelaspisella*. Using these additional data, the genus *Laelaspisella* is redefined more precisely.

## Materials and methods

Samples were collected from bark of elm trees over a period of two years (2002–2004), in Isfahan Province. Mites were removed from the bark by extraction using Tullgren funnels. Mites were cleared in Nesbitt’s solution and mounted in Hoyer’s medium (Walter and Krantz 2009). The line drawings and examinations of the specimens were performed with an Olympus BX51 phase contrast microscope equipped with a drawing tube. All measurements in the descriptions are given in micrometres (µm). Dorsal shield length and width were taken from the anterior to posterior margins along the midline, and at its broadest point, respectively. Length and width of the sternal shield were measured from the anterior point to the posterior point at the full length and broadest point, respectively. Genito-ventral shield length and width were measured along the midline from the posterior margin of the sternal shield to the posterior margin of the genito-ventral shield, and at the maximum, respectively. Leg lengths were measured from base of the coxa to the apex of the tarsus, excluding the pre-tarsus. Lengths for the fixed and movable cheliceral digits were taken from the base of the digits to their tips. The nomenclature used for the dorsal idiosomal chaetotaxy is that of Lindquist and Evans (1965), the leg chaetotaxy is that of Evans (1963), and names of other anatomical structures mostly follow Evans and Till (1979). We use the term “lyrifissures” to refer to slit-shaped sensilli, and “pore” for circular or oval-shaped cuticular openings of unspecified function. Holotype and paratypes of the new species are deposited in the Acarological collection, Department of Plant Protection, Yazd Branch, Islamic Azad University (YIAU); one paratype is deposited in the Jalal Afshar Zoological Museum, College of Agriculture, University of Tehran, Iran (JAZM) and one paratype is also deposited in the Australian National Insect Collection, CSIRO Ecosystem Sciences, Canberra, Australia (ANIC).

## Taxonomy

### Genus Laelaspisella

Taxon classificationAnimaliaMesostigmataLaelapidae

Marais & Loots, 1969

Laelaspisella Marais & Loots, 1969: 1.

#### Type species.

*Laelaspisella epigynalis* Marais & Loots, 1969, by original designation.

#### Notes on the genus.

The presence of pre-sternal plates and an expanded epigynal shield suggests a superficial similarity to *Gymnolaelaps*. However, *Laelaspisella* has a hypertrichous dorsal shield, two ventral setae on genu IV, and lacks metasternal setae *st4*. *Gymnolaelaps* has a normal complement of 40 pairs of setae on the dorsal shield, one ventral seta on genu IV, and the metasternal setae are always present.

#### Diagnosis.

The genus is characterised by a well sclerotised hypertrichous holodorsal shield, (podonotal area hypertrichous or with normal chaetotaxy), convex dorsal shield and flat venter, and a large genito-ventral shield, expanded posterior to the genital setae, with strong reticulated ornamentation. Pre-sternal plates present (lightly sclerotised in the new species); female sternal shield deeply concave in posterior margin and lateral corners extended to the level of coxa III, with three pairs of simple sternal setae; endopodal shields between coxae II and III fused with sternal shield. Metasternal setae *st4* always absent; pores iv3 present on the posterolateral extensions of sternal shield; exopodal plate behind coxa IV triangular, more or less contiguous with but separate from peritrematal shields; peritrematal shield extending posteriorly well past coxae IV; genito-ventral shield with rounded posterior margin separate from anal shield, or with straight posterior margin touching anal shield; opisthogastric membrane with eight to nine pairs of smooth setae (r6 is not included), setae *Jv5* and *Zv5* longer than other opisthogastric area setae or normal (not longer than the other dorsal setae); anterior margin of epistome smooth or with irregular minute denticulation; chelicera with small and robust digits with few teeth, dorsal seta sometimes absent. Hypostomal groove with four to six rows of denticles. Corniculi well-sclerotised; palp tarsal claw with two pointed tines. Legs shorter than idiosoma, genu IV with ten setae (2 2/1 3/1 1), genu I with seta *pd3* absent (2 3/2 2/1 2) or present (2 3/2 3/1 2).

These characters are variable within the genus *Laelaspisella*: (1) dorsal seta of chelicera present or absent; (2) podonotal shield hypertrichous or with normal chaetotaxy; (3) setae *Jv5* and *Zv5* expanded or normal; (4) seta *pd3* on genu I present or absent; (5) extra opisthogastric setae present or absent; (6) genito-ventral shield with rounded posterior margin separate from anal shield, or with straight posterior margin touching anal shield; (7) Anterior margin of epistome smooth or with irregular minute denticulation.

To separate *Laelaspisella* from *Gymnolaelaps*, the following characters can be used: *Laelaspisella* has (1) opisthonotal area of dorsal shield hypertrichous; (2) metasternal setae absent; (3) genu IV with two ventral setae; (4) pore iv3 on sternal shield. Gymnolaelaps has (1) opisthonotal area of dorsal shield not hypertrichous; (2) metasternal setae present; (3) genu IV with one ventral seta; (4) pore iv3 in soft skin.

### Results

#### Laelaspisella elsae sp. n.

Taxon classificationAnimaliaMesostigmataLaelapidae

http://zoobank.org/E5F8EAC9-F6EF-4242-9B3C-BBACA0A9B0DB

Figures 1–5 , 6–9

##### Type material.

Holotype, female, Iran, Isfahan, March-April 2002, A. Jalalizand coll., from bark of elm trees (in YIAU). Paratypes, five females same data as holotype (in YIAU, JAZM and ANIC).

##### Description of the female.

**Figures 1–5.**
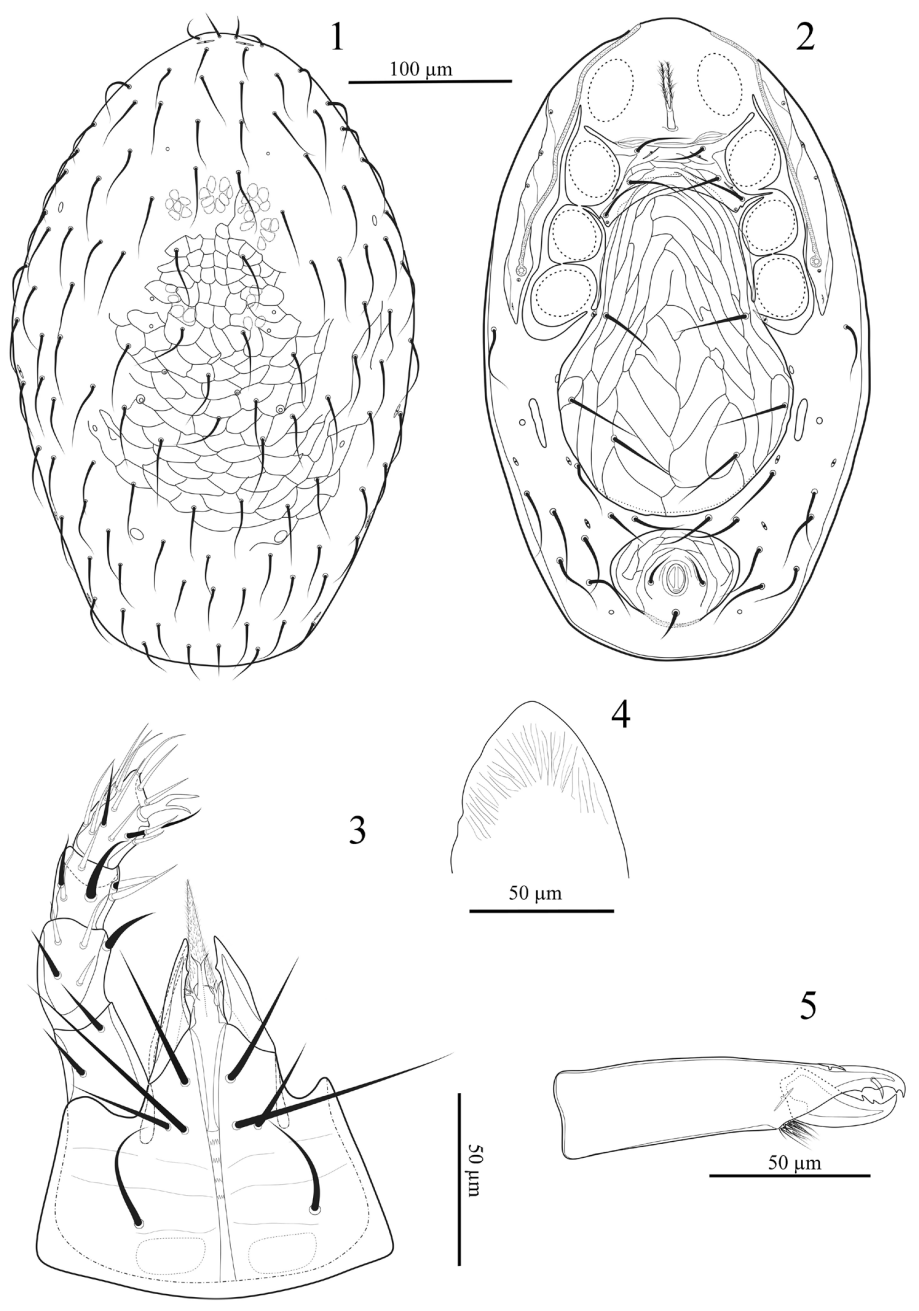
*Laelaspisella elsae* sp. n., female. **1** Dorsal shield **2** Ventral idiosoma **3** Hypostome **4** Epistome **5** Chelicera.

*Dorsal idiosoma* (Fig. 1). Dorsal shield length 400–449, width 281–333 (n = 6). Shield oval shaped, convex, well-sclerotised, reticulated; with about 109–111 simple and long setae, with some unpaired and asymmetrical setae in opisthonotal area, setae similar in length (30–40) and thickness, most long enough to reach well past base of next posterior seta, except *j1* and *z1* (13–15) and some posterio-lateral setae (14–16). Shield with 12 pairs of pore-like structures, apparently including three pairs of gland pores and eight pairs of poroids; lyrifissures near the base of *z1* large and slit-like, others smaller and ovoid.

*Ventral idiosoma* (Fig. 2). Tritosternum with paired pilose laciniae (33–36), columnar base (12–13 × 5–6 wide); presternal area with transverse lightly sclerotised presternal lines. Sternal shield (length 35–43) narrowest between coxae II (99–100), widest at level between coxae II and III (120–128), with convex anterior margin; posterior margin deeply concave; shield bearing three pairs of smooth pointed setae (*st*1 27–32, *st*2 35–40, *st*3 40–43) and two pairs of lyrifissures, one pair adjacent to setae *st1* and the other between *st*2 and *st*3; surface with distinct reticulate ornamentation. Metasternal setae *st4* apparently absent but metasternal poroids present on the posterolateral extensions of sternal shield; endopodal plates II/III fused to sternal shield, endopodal plates III/IV elongate, narrow, curved, but not fused to large triangular podal shields posterior to coxae IV. Genito-ventral shield broad, length 258–267, maximum width 188–195, posterior edge rounded, reticulate throughout, bearing genital setae *st5* (50–55) and two additional pairs of setae on its surface, *Jv*1, *Zv1* (50–62), paragenital poroids present. Anal shield oval, large (64–73 × 82–88 wide), reticulate throughout, anal pores indistinct, para-anal setae (12) shorter than post-anal seta (20), cribrum relatively narrow. Opisthogastric skin with eight pairs of smooth setae (55–65) and four pair of pores; elongate metapodal plates (34–37 × 9–11) close to genito-ventral shield. Peritreme extending from mid-coxa IV to anterior level of coxa I, peritrematal shield wide, with a very wide outer margin, bearing three pairs of discernible pore-like structures, two pairs of poroids opposite coxae II–III and another pair opposite coxae I–II.

*Gnathosoma*. Hypostomal groove with four rows of denticles each bearing 2–5 small teeth (Fig. 3). Corniculi robust and hornlike. Internal malae complex, with two pairs of lobes, inner lobes narrow and long, with smooth edges, outer lobes very short, narrow, branched. Hypostome with four pairs of setae, internal posterior hypostomal setae *h3* longest (67–70), *h1* (42–45), *h2* (28–30), palpcoxal *pc* (32–35) (Fig. 3). Palp chaetotaxy: trochanter 2, femur 5, genu 6, tibia 14, tarsus 15, all setae smooth and needle-like; palp tarsal claw with two pointed tines of equal length, without any hyaline membranes (Fig. 3). Epistome somewhat subtriangular, with a smooth margin (Fig. 4). Fixed digit (40–41) of chelicera with two small pointed teeth (Fig. 5); pilus dentilis moderately thick, dorsal seta not detected, movable digit (36-38) with two large teeth; arthrodial membrane with a row of short filaments.

**Figures 6–9.**
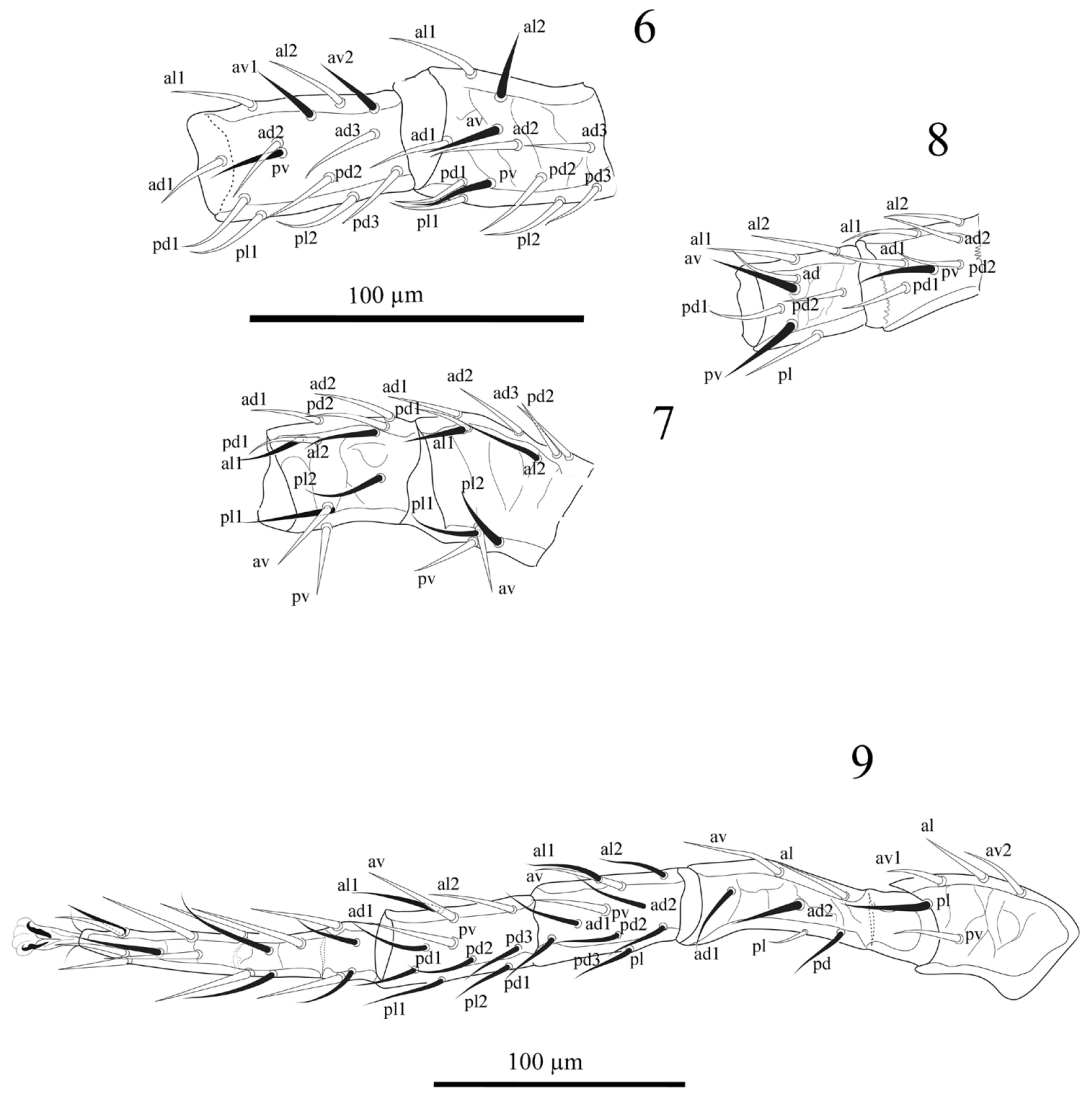
*Laelaspisella elsae* sp. n., female. **6** Genu and tibia I (dorsal aspect) **7** Genu and tibia II (dorsal aspect) **8** Genu and tibia III (dorsal aspect) **9** Leg IV (ventral aspect).

*Legs*. Legs II and III shorter (309-320, 302-310), I and IV longer (349-360, 431-447) (excluding pre-tarsus). Leg I: coxa 0 0/1 0/1 0, trochanter 1 0/2 1/1 1, femur 2 2/1 3/3 2, genu 2 3/2 3/1 2 (Fig. 6), tibia 2 3/2 3/1 2 (Fig. 6). Leg II: coxa 0 0/1 0/1 0, trochanter 1 0/2 0/1 1, femur 2 3/1 2/2 1, genu 2 3/1 2/1 2 (Fig. 7), tibia 2 2/1 2/1 2 (Fig. 7). Leg III: coxa 0 0/1 0/1 0, trochanter 1 0/2 0/1 1, femur 1 2/1 1/0 1, genu 2 2/0 2/1 0 (Fig. 8), tibia 2 1/1 2/1 1 (Fig. 8). Leg IV (Fig. 9): 0 0/1 0/0 0, trochanter 1 0/2 0/1 1, femur 1 2/1 1/0 1, genu 2 2/1 3/1 1, tibia 2 1/1 3/1 2; all setae fine and needle-like. Tarsi I-IV with 18 setae 3 3/2 3/2 3 + *mv*, *md.* All pre-tarsi with a pair of claws and a long thin membranous ambulacral stalk.

##### Insemination structures.

Not seen, apparently unsclerotised.

##### Etymology.

It is with great pleasure that we name this species after Elsa Joharchi, the new-born daughter of the first author.

##### Remarks.

*Laelaspisella elsae* sp. n. differs from all other species in the genus in having the genito-ventral shield broad and bearing genital setae *st5* and two additional pairs of setae on its surface, hypostomal groove with four rows of denticles, genu I with seta *pd3* (2 3/2 3/1 2) and two ventral setae on genu IV (2 2/1 3/1 1), also dorsal seta of chelicera absent.

#### Laelaspisella tonsilis

Taxon classificationAnimaliaMesostigmataLaelapidae

(Karg, 1989) comb. n.

Pseudoparasitus (Gymnolaelaps) tonsilis Karg, 1989a: 335.

##### Specimens examined.

The paratype specimen of Karg’s collection was examined by the first author and this information is as follows: Slide ZMB Kat. Nr. 41473, labeled *Pseudoparasitus tonsilis* Karg, 1989a, Nr. 3942♀, paratypus, St. Lucia, Antillen, Castries, Vigie point, Eins.: Dr. Mahunka, Budapest, 11.7.80.

##### Remarks.

Pseudoparasitus (Gymnolaelaps) tonsilis shares all important character states with the genus *Laelaspisella* : dorsal shield hypertrichous, two ventral setae on genu IV (2 2/1 3/1 1), genu I (2 3/2 3/1 2) seta *pd3* present, lacks metasternal setae *st4*, seta *Jv5* long (Karg 1989a in his description named this seta as *Z5*). Therefore we consider Pseudoparasitus (Gymnolaelaps) tonsilis as a member of *Laelaspisella*.

#### Laelaspisella berlesei

Taxon classificationAnimaliaMesostigmataLaelapidae

Joharchi nom. n.

Laelaps (Eulaelaps) canestrinii Berlese 1903: 13.
Laelaps (Hypoaspis) canestrinii Berlese 1904: 412.
Gymnolaelaps canestrinii (Berlese, 1903) sensu Costa, 1962: 491.

##### Remarks.

The identity of *Laelaspisella canestrinii* is very confused. In *Laelaps canestrinii* Berlese, 1892, the female has a very wide genito-ventral shield carrying four pairs of setae in addition to *st5*, and has a straight posterior margin. There are no setae between the genito-ventral and anal shields. The sternal shield has only two pairs of setae, the metasternal plates and setae are absent, the anal shield is wider than long, and the movable digit of the chelicera has three teeth. In the male the anal shield is fused to the genito-ventral shield, with the fusion marked by a distinct line. Berlese (1903) referred to this species as Laelaps (Eulaelaps) canestrinii. Berlese (1904) then added some morphological information and illustrations for a species that he called Laelaps (Hypoaspis) canestrinii. In these illustrations the genito-ventral shield of the female carries only one pair of setae and has a rounded posterior margin. The anal shield is narrow, and there is a pair of setae between the genito-ventral shield and the anal shield. In the male, the anal shield is clearly separate from the genito-ventral shield. These descriptions appear to refer to two different species. Hunter (1967) referred to this problem but did not resolve it. *Laelaps canestrinii* does not belong to the genera *Laelaps* or *Hypoaspis*, and a solution to the identification of the true genus of *Laelaps canestrinii* Berlese, 1892 can only come from a detailed study of Berlese’s specimens. The 1904 re-description is only a misidentification of the 1892 species. Costa (1962) re-described and illustrated a species he called *Gymnolaelaps canestrinii* (Berlese, 1903), but he did not mention *Laelaps canestrinii* Berlese, 1892. Costa was wrong about this species because only the 1892 description and illustrations refer to the true species of *canestrinii*. Therefore *Laelaps canestrinii* *sensu* Berlese (1903), (1904) and Costa (1962) does not have a name. Therefore, we rename this species as *Laelaspisella berlesei* Joharchi, nom. n. (=Laelaps (Eulaelaps) canestrinii Berlese, 1903 = Laelaps (Hypoaspis) canestrinii Berlese, 1904 = *Gymnolaelaps canestrinii* (Berlese, 1903) *sensu* Costa, 1962) in honour of Antonio Berlese. In view of this confusion, it is difficult to determine the identity of the specimens cited under these names by other authors.

## Discussion

All six species of *Laelaspisella* share four important diagnostic character states: (1) hypertrichous dorsal shield (in both the podonotal and opisthonotal region or only in the opisthonotal region); (2) two ventral setae on genu IV (2 2/1 3/1 1); (3) metasternal setae *st4* absent but metasternal poroids present on the posterolateral extensions of sternal shield; (4) Palp tarsal claw with two pointed tines.

Some of the diagnostic characters of the *Laelaspisella elsae* were unique within the known *Laelaspisella* species (such as: presternal area with transverse lightly sclerotised presternal lines, genito-ventral bearing genital setae *st5* and two additional pairs of setae on its surface and hypostomal groove with four rows of denticles each bearing 2-5 small teeth) but at the present time, creating a new monotypic genus to accommodate the new species would not help to clarify the taxonomic problems existing within the family Laelapidae. Therefore, this species is provisionally placed in *Laelaspisella* until a comprehensive revision of all these genera resolves its relationships.

The key below distinguishes the six species of *Laelaspisella*. In this key we recognise two distinct groups of species within the genus. All species group of *epigynalis* have 12 setae on genu I (2 3/2 2/1 2), with seta *pd3* absent. Group *elsae* species have 13 setae on genu I (2 3/2 3/1 2), with seta *pd3* present.

### Key to species of *Laelaspisella*

**Table:** 

1	Genu I with 12 setae (2 3/2 2/1 2) *pd3* absent *epigynalis* species group	**2**
–	Genu I with 13 setae (2 3/2 3/1 2) *pd3* present *elsae* species group	**3**
2	Genito-ventral shield tapered posteriorly, opisthogastric area with eight pairs of setae	***Laelaspisella macrodorsalis* Marais & Loots, 1969**
–	Genito-ventral shield rounded posteriorly, opisthogastric area with nine pairs of setae	***Laelaspisella epigynialis* Marais & Loots, 1969**
3	Dorsal shield hypertrichous in both the podonotal and opisthonotal region	**4**
–	Dorsal shield hypertrichous only in the opisthonotal region	**5**
4	Post-stigmatal section of peritrematal shield elongate, extending well behind exopodal shield	***Laelaspisella tonsilis* (Karg, 1989a)**
–	Post-stigmatal section of peritrematal shield short and wide	***Laelaspisella kabitae* (Bhattacharyya, 1968)**
5.	Genito-ventral shield bearing genital setae *st5* and two additional pairs of setae on its surface, dorsal shield with long setae	***Laelaspisella elsae* sp. n.**
–	Genito-ventral shield bearing only genital setae *st5* on its surface, dorsal shield with short setae	***Laelaspisella berlesei* Joharchi, nom. n.**

## Supplementary Material

XML Treatment for Laelaspisella

XML Treatment for Laelaspisella elsae

XML Treatment for Laelaspisella tonsilis

XML Treatment for Laelaspisella berlesei
